# Low Prevalence of *CHEK2* Gene Mutations in Multiethnic Cohorts of Breast Cancer Patients in Malaysia

**DOI:** 10.1371/journal.pone.0117104

**Published:** 2015-01-28

**Authors:** Suriati Mohamad, Nurismah Md Isa, Rohaizak Muhammad, Nor Aina Emran, Nor Mayah Kitan, Peter Kang, In Nee Kang, Nur Aishah Mohd Taib, Soo Hwang Teo, Sharifah Noor Akmal

**Affiliations:** 1 Department of Pathology, Faculty of Medicine, Universiti Kebangsaan Malaysia Medical Centre, Cheras, Kuala Lumpur, Malaysia; 2 Department of Surgery, Faculty of Medicine, Universiti Kebangsaan Malaysia Medical Centre, Cheras, Kuala Lumpur, Malaysia; 3 Department of General Surgery, Hospital Kuala Lumpur, Kuala Lumpur, Malaysia; 4 Department of Endocrine Surgery, Hospital Putrajaya, Putrajaya, Malaysia; 5 Cancer Research Initiative Foundation (CARIF), Subang Jaya, Selangor, Malaysia; 6 University Malaya Cancer Research Institute, Faculty of Medicine, University Malaya, Kuala Lumpur, Malaysia; CNR, ITALY

## Abstract

*CHEK2* is a protein kinase that is involved in cell-cycle checkpoint control after DNA damage. Germline mutations in *CHEK2* gene have been associated with increase in breast cancer risk. The aim of this study is to identify the *CHEK2* gene germline mutations among high-risk breast cancer patients and its contribution to the multiethnic population in Malaysia. We screened the entire coding region of *CHEK2* gene on 59 high-risk breast cancer patients who tested negative for *BRCA1/2* germline mutations from UKM Medical Centre (UKMMC), Hospital Kuala Lumpur (HKL) and Hospital Putrajaya (HPJ). Sequence variants identified were screened further in case-control cohorts consisting of 878 unselected invasive breast cancer patients (180 Malays, 526 Chinese and 172 Indian) and 270 healthy individuals (90 Malays, 90 Chinese and 90 Indian). By screening the entire coding region of the *CHEK2* gene, two missense mutations, c.480A>G (p.I160M) and c.538C>T (p.R180C) were identified in two unrelated patients (3.4%). Further screening of these missense mutations on the case-control cohorts unveiled the variant p.I160M in 2/172 (1.1%) Indian cases and 1/90 (1.1%) Indian control, variant p.R180C in 2/526 (0.38%) Chinese cases and 0/90 Chinese control, and in 2/180 (1.1%) of Malay cases and 1/90 (1.1%) of Malay control. The results of this study suggest that *CHEK2* mutations are rare among high-risk breast cancer patients and may play a minor contributing role in breast carcinogenesis among Malaysian population.

## Introduction

Cell cycle checkpoint kinase 2 gene, (*CHEK2*) (OMIM +604373) encodes a checkpoint serine/threonine kinase and is the human homolog of *Saccharomyces cerevisiae RAD53* and *S*. *pombe CDS1* [1]. The *CHEK2* gene is critical in DNA damage induced cell-cycle checkpoint control by activating *P53* and *BRCA1*, which in turn play an important role in controlling the cell cycle checkpoints, apoptosis and DNA damage repair [2].

The *CHEK2* gene has been proposed as a moderate penetrance breast cancer susceptibility gene [3]. However, germline *CHEK2* mutations are rare and are largely population-specific. Among *BRCA*1/2-negative familial breast cancer patients, the prevalence of different *CHEK2* germline mutations was reported with the frequency of 0% (0/25) in the French-Canadian population [4], 2.9% (15/507) in France [5], 5.8% (30/516) in Germany [6], 5.8% (10/172) in Ashkenazi Jewish population [7], 8.9% (8/89) in the UK, North America and the Netherlands [8] and 12.2% (10/82) in Finland [9]. Among early onset breast cancer patients, the mutations were reported with the frequency of 3% (5/169) in multi-ethnic population from US [10] and 3.9% (51/1303) in United States, Canada and Australia [11].

The most extensively studied *CHEK2* protein truncating mutation, c.1100delC was reported to confer a two-fold increased breast cancer risk among Dutch population [3]. The *CHEK2* c.1100delC is present in 1.4% Nothern European countries including Finland, UK and the Netherlands [3,12–13] and was absent in South America, Spain and all Asian populations studied so far including India, Japan, China, Korea, Singapore, the Philippines, Pakistan and Malaysia [14–22].

In addition, four other *CHEK2* variants have been associated with increased breast cancer risks including the missense mutations p.I157T and p.S428F, the splice site mutation IVS2 + 1G > A and the large genomic deletion del5395. The missense mutation, p.I157T was reported in higher frequency and associated with increased breast cancer risk among Finnish, Polish and German population with a frequency of 2.2%-7.4% [23–25] and lower in North America, UK, the Netherlands and US [8,10,26]. Another missense mutation, p.S428F was only found in Ashkenazi Jewish breast cancer patients with a frequency of 2.88% [27]. The splice site mutation IVS2 + 1G > A was reported to have a two-fold increased breast cancer risk in Polish population with a frequency of 1.1% and with a low frequency in German and Byelorussian populations [25]. A large genomic deletion of 5,395 bp, which results in loss of exons 9 and 10, was reported to confer a twofold increase in breast cancer risk in Czech, Slovak and Polish populations with a frequency of 0.9–1.3% [28–29]. These mutations have also been reported to increase risk to prostate and colorectal cancer [30–32].

To date, two *CHEK2* prevalence studies have been conducted in Asia which are in China and Pakistan [33–34]. In this study, we screened the mutations on the entire coding sequence of the *CHEK2* gene by direct sequencing on a cohort of Asian high-risk breast cancer patients who have been tested negative for *BRCA1* and *BRCA2* gene mutations. The variants detected by sequencing were further screened in case-control cohorts to determine the prevalence of the variants and its contribution in multi-ethnic population in Malaysia.

## Materials and Methods

### Study subjects

a) High-risk breast cancer patients for sequencing

A total of 59 high-risk breast cancer patients who tested negative for *BRCA1* and *BRCA2* mutations were included in the study. The recruitment of breast cancer patients for this study was done in three main breast clinics in the Klang Valley; UKM Medical Centre (UKMMC), Hospital Kuala Lumpur (HKL) and Hospital Putrajaya (HPJ). Each patient who presented with either one or a combination of the criteria set below was approached and given a consent form for the use of clinical records and blood specimens were collected. The inclusion criteria for *CHEK2* testing are: 1) early-onset of breast cancer (≤ 40 years); 2) breast cancer patients with at least one or more family member (s) with breast and/ or ovarian cancer in first-, second- or third-degree relatives; 3) bilateral breast cancer and 4) male patients with breast cancer. Information regarding nature and objectives of the study had been explained to the patients and informed consent was taken. Each patient was interviewed to obtain data on family history and family pedigree. Ten milliliters of peripheral blood specimen from each of the selected patient was collected in EDTA tubes.

b) Breast cancer cases and controls for genotyping

The recruitment of breast cancer patients to the Malaysian Breast Cancer Genetic Study started in January 2003 at the University Malaya Medical Centre and included both prevalent and incident breast cancer cases. In the present study, blood samples from 880 patients diagnosed with invasive breast cancer were available for genotyping to determine the prevalence of *CHEK2* variants and of these, 878 samples were successfully genotyped. There were 526 Chinese (59.9%), 180 Malays (20.5%), and 172 Indians (19.6%). In addition, 270 healthy individuals (90 Malays, 90 Chinese and 90 Indian) attending the diagnostic centre for non-cancer related health concerns in University Malaya Medical Centre were included in the case-control study.

### Ethics statement

All study subjects provided written informed consent. Blood, demographic and family history data were collected from breast cancer patients who consented to participate in this study. The study was approved by the Medical Ethics Committee of Universiti Kebangsaan Malaysia Medical Centre (UKMMC).

### Mutation detection

Genomic DNA from blood was extracted using QIAamp DNA Midi Kit (QIAGEN, Germany) according to the manufacturer’s protocol. Fourteen primer pairs of *CHEK2* were applied to amplify 14 coding exons and intron-exon boundaries of the *CHEK2* gene using AmpliTaq Gold DNA Polymerase (Applied Bioscience, USA). Because multiple copies of *CHEK2* pseudogenes exist for exons 10–14, a nested PCR was performed to specifically amplify the chromosome 22 copy of the *CHEK2* gene [35]. Long range pre-amplification of exon 10–14 were employed by Expand Long Template PCR System (Roche Applied Science, Mannheim, Germany) according to the manufacturer’s protocol. Products of long range PCR were then used as a template to amplify individual exon 10–14 using appropriate oligonucleotide primers. PCR products were purified using the QIAquick PCR Purification kit (QIAGEN, Germany). Subsequently, direct DNA sequencing was carried out to detect the presence of sequence alterations using the ABI BigDye Terminator kit (version 3.1) on 3130xl Sequencer Analyzer (Applied Biosystem, Foster City, CA).

### Genotyping analysis

Multiplex genotyping of two *CHEK2* missense mutations identified in exon 3 were performed using high throughput Sequenom MassARRAY iPLEX platform (Sequenom Inc., San Diego, California, USA). All mutations identified were confirmed by direct DNA sequencing in an independent DNA sample. The detail on the genotyping analysis protocol was described previously [36].

### *In silico* analysis

In this study, the pathogenecity of the missense mutations (p.I160M and p.R180C) was predicted using three computational analyses based on sequence homology and physical properties of amino acids which are Align-GVGD (http://agvgd.iarc.fr/alignments.php), PolyPhen-2 (http://genetics.bwh.harvard.edu/pph/) and SIFT (http://blocks.fhcrc.org/sift/SIFT.html).

## Results

In this study, the entire coding sequence of the *CHEK2* gene was screened for mutation on a total of 59 breast cancer patients who were non-*BRCA* carriers from UKM, HKL and HPJ using direct DNA sequencing. Of these, 37 (62.7%) were Malays, 14 (23.7%) were Chinese and 8 (14.3%) were Indians. The median age of onset of breast cancer was 38 years old (range 17–70). Thirty-nine (66.1%) patients were diagnosed with breast cancer <40 years old, 4 (6.8%) had bilateral breast cancers, 15 (25.4%) had a positive family history and one (1.7%) was a male breast cancer patient (Table 1).

**Table 1:** Characteristics of high risk breast cancer patients selected for *CHEK2* mutation screening according to ethnicity, n = 59.

Ethnicity	Malay	Chinese	Indian	Total
	No (%)	No (%)	No (%)	No (%)
Total	37 (62.7)	14 (23.7)	8 (13.6)	59
Female breast cancer	37	13	8	58 (98.3)
Male breast cancer	0	1	0	1 (1.7)
Age at diagnosis				
<30	9	0	0	9 (15.3)
31–40	19	7	5	31 (52.5)
41–50	2	2	2	6 (10.2)
51–60	6	2	1	9 (15.3)
>60	1	3	0	4 (6.8)
Personal cancer history				
Bilateral breast cancer	3	2	1	6 (10.2)
Ovarian cancer	1	0	0	1 (1.7)
Family history of breast and/or ovarian cancer (up to 3rd degree relatives)				
0 case	13	5	5	23 (39.0)
1 case	20	6	2	28 (47.5)
2 cases	2	1	3	6 (10.2)
3 cases	1	0	0	1 (1.7)
>3 cases	1	0	0	1 (1.7)

### a) Mutation analysis

Mutational analyses of the entire coding sequence of *CHEK2* revealed four sequence variants consisting of two missense mutations detected in exon 3 and two silent mutations in exon 1 and exon 2. Both missense mutations p.I160M and p.R180C were detected in two unrelated patients (3.4%). The *CHEK2* p.I160M missense mutation was detected in a Malay patient who was diagnosed with breast cancer at the age of 43 years old. She had grade 1 infiltrating ductal carcinoma with estrogen receptor (ER) and progesteron receptor (PR) status positive while negative for HER-2 receptor (Table 2). The patient had a mother who was diagnosed with breast cancer at an unknown age.

**Table 2:** Characteristics of the *CHEK2* gene mutation carriers identified from sequencing and genotyping cohorts.

Exon	Nucleotide change (HGVS)	AA change	Cohort	Ethnicity	Diagnosis, Age	Histology	Grade	ER	PR	HER2
3	c.480A>G	p.I160M	Sequencing	Malay	Breast cancer, 43	IDC	1	+	+	-
			Genotyping	Indian	Breast cancer, 52	IDC	1	+	+	1+
				Indian	Breast cancer, 46	IDC	2	-	-	3+
3	c.538C>T	p.R180C	Sequencing	Malay	Breast cancer, 15	IDC	3	-	-	1+
					Bilateral ovarian cancer, 14	Granulosa Cell Tumour	NA	NA	NA	NA
			Genotyping	Malay	Breast cancer, 41	IDC	2	-	NA	-
				Malay	Breast cancer, 43	IDC	2	-	-	+
				Chinese	Breast cancer, 45	IDC	1	-	-	-
				Chinese	Breast cancer, 33	NA	NA	NA	NA	NA

AA: Amino acid, IDC: infiltrating ductal carcinoma, ER: estrogen receptor, PR: progesteron receptor, HER2: Human epidermal growth factor 2 receptor, −: negative, +: positive, NA: not available.

**Fig 1:**
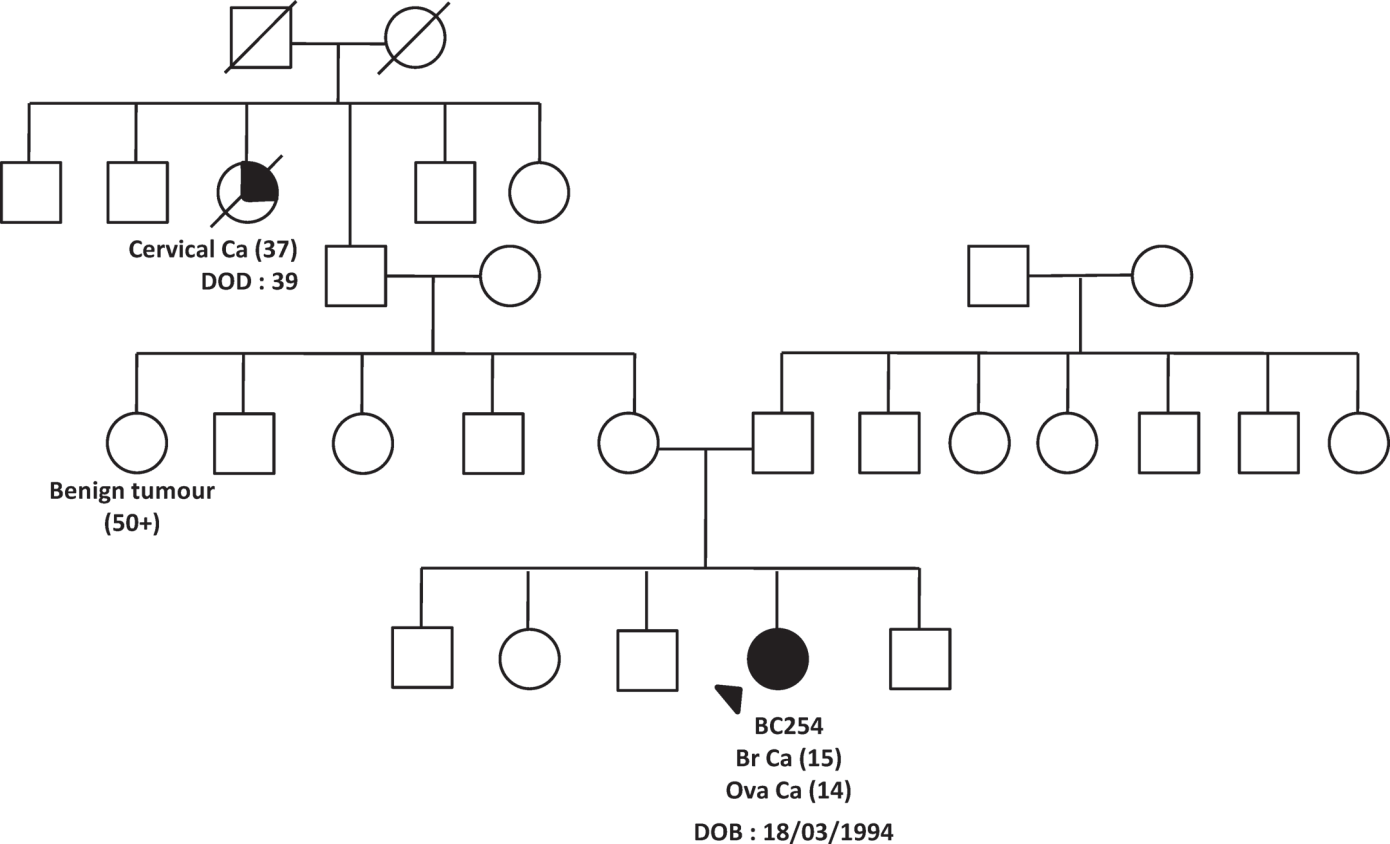
Family pedigree of *CHEK2* p.R180C missense mutation’s carrier (BC254) from sequencing analysis. Index patients are indicated with an arrow while individuals affected with breast cancer are indicated with filled symbol. Date of birth and age of diagnosis (in bracket) for affected individual are indicated. Deceased individuals are indicated with a slash.

Another missense mutation, *CHEK2* p.R180C was detected in a Malay patient who was diagnosed with early onset breast cancer, at the age of 15 years old. The patient had high grade (grade 3) infiltrating ductal carcinoma with estrogen and progesterone receptor negative and HER-2 status positive (Table 2). Prior to the breast cancer, she was diagnosed with bilateral granulosa germ cell tumour of the ovaries stage IV with liver metastases at the age of 14 years and had undergone TAHBSO. She had a family history of cervical cancer from her maternal grand aunt who was diagnosed with the cancer at the age of 37 years (Fig. 1).

Besides that, one synonymous mutation in exon 1, c.252A>G (p.E84E) was detected in 10 patients (16.9%). This variant has been previously reported in several other *CHEK2* studies and is likely to be a neutral polymorphism with no elevated risk of breast cancer. A novel synonymous mutation in exon 2, c.387G>A (p.L129L) was detected in one patient (1.7%). This mutation is unlikely to have association with the disease due to the silent amino acid changes.

### b) Case-control analysis

**Fig 2:**
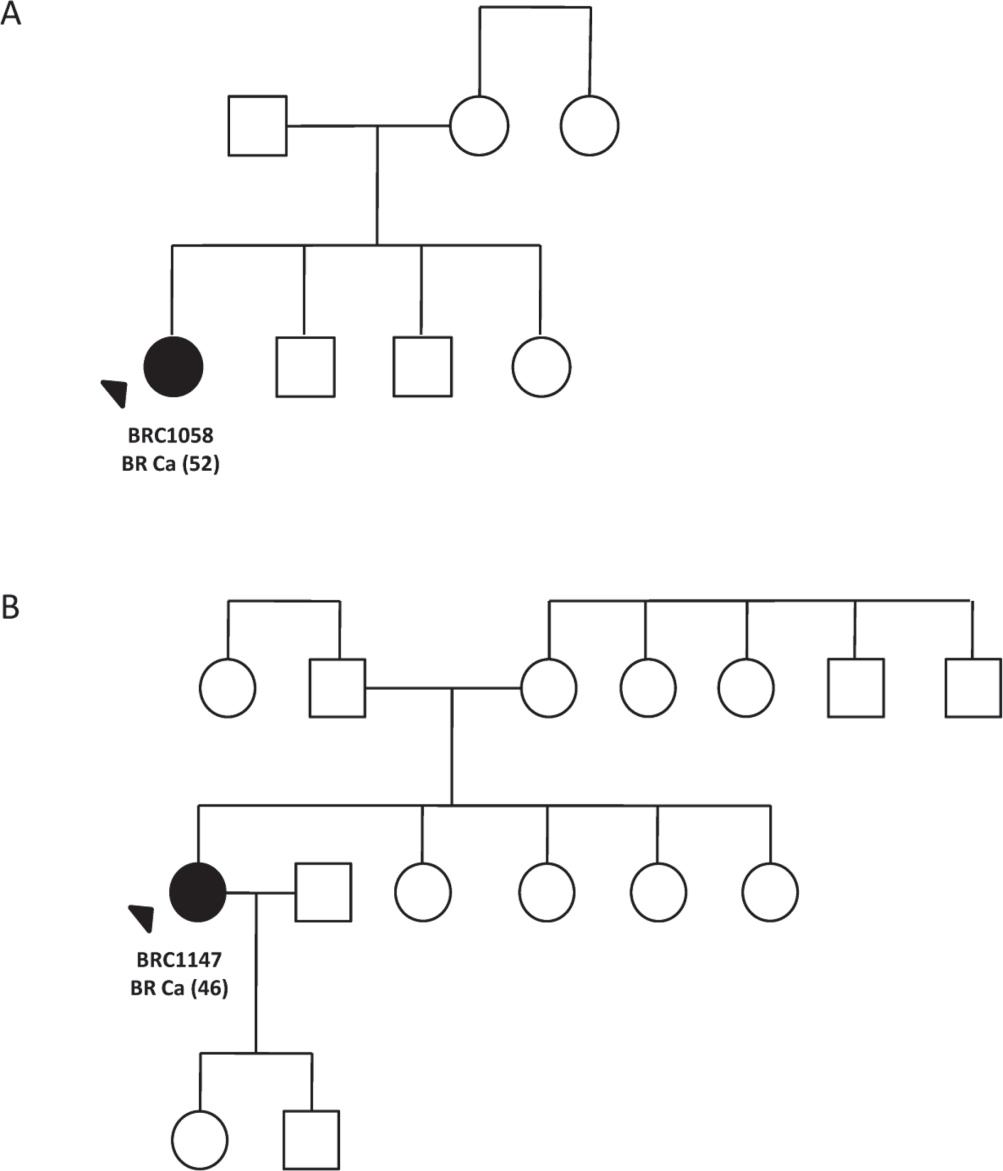
Family pedigree of *CHEK2* p.I160M missense mutation’s carriers in case-control analysis; (A) family pedigree of BRC1058 and (B) BRC1147. Index patients are indicated with an arrow while individuals affected with breast cancer are indicated with filled symbol. Age of diagnosis (in bracket) for affected individuals is indicated. Deceased individuals are indicated with a slash.

**Fig 3:**
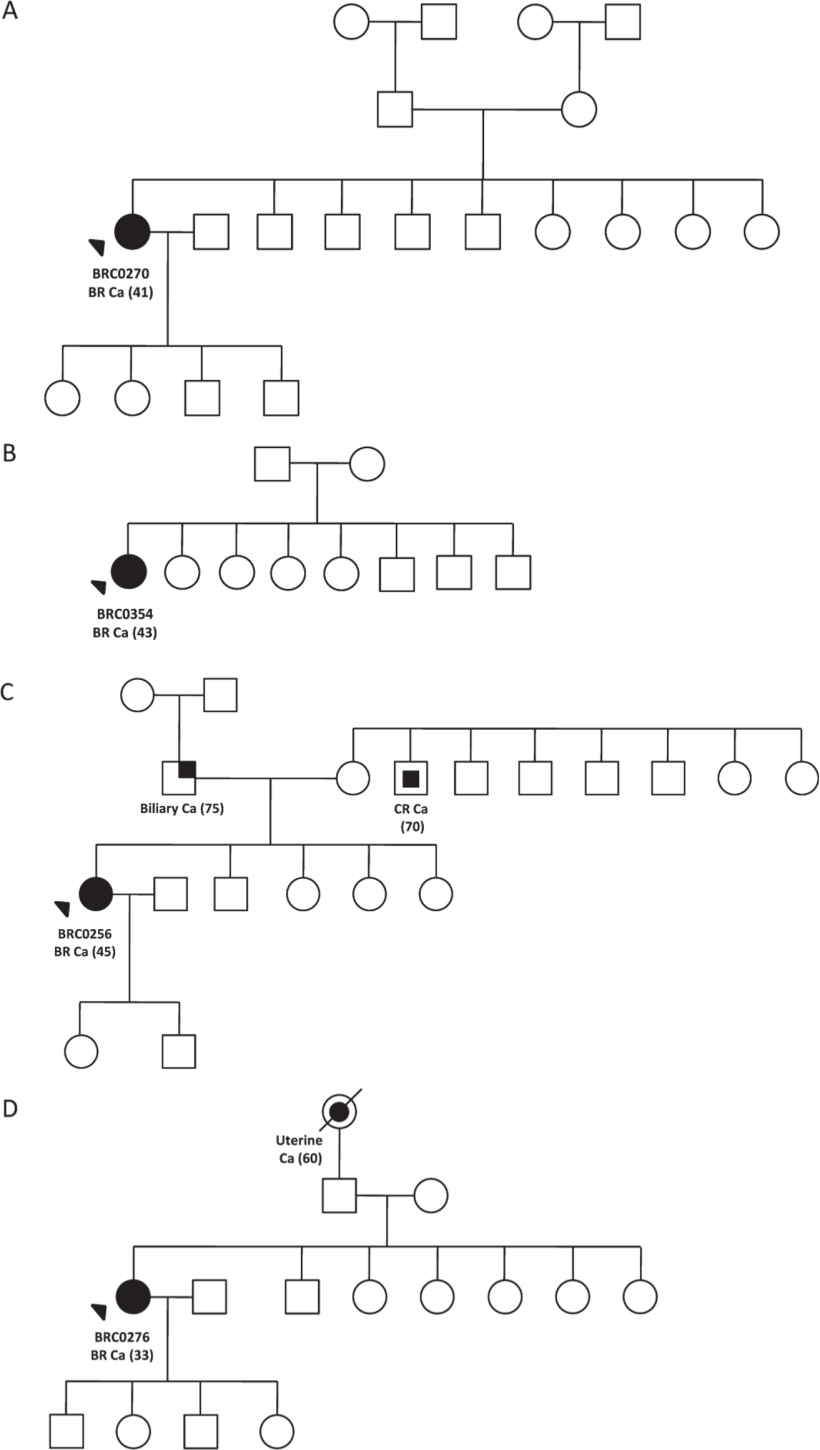
Family pedigree of *CHEK2* p.R180C missense mutation’s carriers from case-control analysis; (A) BRC256, (B) BRC276, (C) BRC270 and (D) BRC354. Index patients are indicated with an arrow while individuals affected with breast cancer are indicated with filled symbol. Date of birth and age of diagnosis (in bracket) for affected individual are indicated. Deceased individuals are indicated with a slash.

Further analysis in a case-control cohort unveiled *CHEK2* p.I160M variant in 2/172 (1.16%) Indian patients and 1/90 (1.11%) Indian control while p.R180C variant was found in 2/180 (1.11%) Malay patients and 1/90 (1.1%) Malay controls, and in 2/526 (0.38%) Chinese patients and 0/90 Chinese controls (p = 0.98). Notably, both Indian cases carrying the p.I160M variant were diagnosed with breast cancer at the age >45 while all four *CHEK2* p.R180C carriers were diagnosed with breast cancer at the age of ≤45 (Table 2). Both Indian cases (Fig. 2A & 2B) carrying the p.I160M variant and Malay cases (Fig. 3A & 3B) carrying p.R180C variant have no family history of any cancers whilst both Chinese carriers (Fig. 3C & 3D) for p.R180C variant have a history of other cancers in the family such as biliary, colorectal and uterine cancer. Both variants were predicted to be possibly damaging by SIFT and Polyphen-2 analysis.

## Discussion

In this study, the entire coding sequence of the *CHEK2* gene was screened for mutation on 59 breast cancer patients who were non-*BRCA1* and *BRCA2* mutation carriers and two previously reported missense mutations, p.I160M and p.R180C were identified in two unrelated breast cancer patients. Further screening on Malay, Chinese and Indian cases and control cohorts showed these missense mutations were found in both cases and controls suggesting that these variants may not contribute significantly to genetic predisposition to breast cancer in the Malaysian population.

Notably, none of the subjects in this study carried the *CHEK2* c.1100delC germline mutation. Our results with regards the absence of the c.1100delC mutation are consistent with previous studies done on other Asian populations which indicate no contribution of c.1100delC mutation to breast cancer susceptibility in Asian populations [16–22].

The *CHEK2* p.I160M missense mutation was previously reported in 1/516 (0.2%) German familial breast cancer patients [6]. Since the p.I160M variant is located in the forkhead-associated (FHA) domain of the *CHEK2* protein, which is important for dynamic interactions between upstream regulators or downstream targets of the *CHEK2* [37], this mutation could lead to the deregulation of *CHEK2* or structural abnormalities which may hinder protein-protein interactions. Notably, *in silico* analysis (SIFT and Polyphen-2) and an *in vitro* yeast-based assay suggested that this variant may have intermediate response to DNA damage [38].

The *CHEK2* p.R180C was previously reported in 0.58% (3/516) German familial breast cancer patients and 0.2% (1/500) controls [6], 0.58% (1/172) high-risk Ashkenazi Jewish breast/and or ovarian cancer families [7], 0.84% (1/118) familial Chinese breast cancer patients who were non-*BRCA1*/2 carriers [33], and in 0/673 Czech breast cancer patients and 0.15% (1/683) controls [39]. The *CHEK2* p.R180C variant was also previously reported in 2.1% (2/94) prostate tumor samples of Caucasian American patients who were diagnosed before the age of 59 and in germline DNA of 1/423 (0.24%) unaffected men [31]. Another variant in the same amino acid position (the p.R180H variant) was previously reported in a young woman with bilateral female breast cancer who also carried the p.1100delC variant and in an individual with sporadic prostate cancer (1/400, 0.25%) [31,40]. Although this residue is not located within a functional domain, the mutation in this region may affect the structure or stability of FHA elements at the FHA-Kinase domain interface since both side chains are implicated in intra-molecular contacts [41]. Notably, whilst the p.R180C variant is predicted to be likely damaging by *in silico* analysis (SIFT and Polyphen-2) and has been demonstrated to have intermediate response to DNA damage in a yeast-based assay, the p.R180H variant is predicted to be likely benign (align GVGD and SIFT), has little effect on protein expression, phosphorylation and stability, but has lower but not null kinase activity [5,38,42].

Taken together, three studies have determined the prevalence of *CHEK2* germline mutations using whole gene screening in Asia [33–34]. In Pakistan, two potentially deleterious missense mutations, novel p.P92R and p.R406C were identified in two unrelated familial breast cancer patients (1.4%, 2/145) who were non-*BRCA* carriers [34]. These missense mutations are rare and are less likely to contribute to breast/ovarian cancer susceptibility in Pakistan since they were not detected in further screening of 229 high-risk breast cancer patients who were non-*BRCA* carriers and 150 controls. In China, a recurrent missense mutation, p.H371Y was reported to confer moderate risk of breast cancer in Chinese women with the frequency of 4.24% (5/118), 1.76% (16/909) and 0.73% (9/1228) in familial, unselected breast cancer cases and controls respectively (p = 0.041) [33]. Our study identified two other variants (p.I90M and p.R180C) but show that these are rare and not significantly associated with an increased risk to breast cancer. However, given the rarity of *CHEK2* variants in the Asian population, further studies and family-based co-segregation analyses are required to determine the cancer risks associated with these variants.

## Conclusion

In summary, two potentially pathogenic missense mutations were found among 59 high-risk breast cancer patients who were non-*BRCA* carriers with the frequency of 3.4%. Further screening in case-control cohorts showed that these variants were found in both cases and controls. The results of this study suggest that *CHEK2* mutations are rare among high-risk breast cancer patients and may play a minor role in genetic predisposition to breast cancer in the Malaysian population.
